# Facile synthesis of PEDOT-rGO/HKUST-1 for high performance symmetrical supercapacitor device

**DOI:** 10.1038/s41598-021-91100-x

**Published:** 2021-06-03

**Authors:** Dharshini Mohanadas, Muhammad Amirul Aizat Mohd Abdah, Nur Hawa Nabilah Azman, Thahira B. S. A. Ravoof, Yusran Sulaiman

**Affiliations:** 1 Department of Chemistry, Faculty of Science, Universiti Putra Malaysia, 43400 UPM Serdang, Selangor Malaysia; 2 Foundry of Reticular Materials for Sustainability (FORMS), Materials Synthesis and Characterization Laboratory, Institute of Advanced Technology, Universiti Putra Malaysia, 43400 Serdang, Selangor Malaysia; 3 Functional Devices Laboratory, Institute of Advanced Technology (ITMA), Universiti Putra Malaysia, 43400 UPM Serdang, Selangor Malaysia

**Keywords:** Energy storage, Materials for devices

## Abstract

A novel poly(3,4-ethylenedioxythiophene)-reduced graphene oxide/copper-based metal–organic framework (PrGO/HKUST-1) has been successfully fabricated by incorporating electrochemically synthesized poly(3,4-ethylenedioxythiophene)-reduced graphene oxide (PrGO) and hydrothermally synthesized copper-based metal–organic framework (HKUST-1). The field emission scanning microscopy (FESEM) and elemental mapping analysis revealed an even distribution of poly(3,4-ethylenedioxythiophene) (PEDOT), reduced graphene oxide (rGO) and HKUST-1. The crystalline structure and vibration modes of PrGO/HKUST-1 were validated utilizing X-ray diffraction (XRD) as well as Raman spectroscopy, respectively. A remarkable specific capacitance (360.5 F/g) was obtained for PrGO/HKUST-1 compared to HKUST-1 (103.1 F/g), PrGO (98.5 F/g) and PEDOT (50.8 F/g) using KCl/PVA as a gel electrolyte. Moreover, PrGO/HKUST-1 composite with the longest charge/discharge time displayed excellent specific energy (21.0 Wh/kg), specific power (479.7 W/kg) and an outstanding cycle life (95.5%) over 4000 cycles. Thus, the PrGO/HKUST-1 can be recognized as a promising energy storage material.

## Introduction

Supercapacitors are electrical devices which have an excellent energy storage system compared to the conventional capacitors as it possesses high specific energy, long-term cycling life as well as rapid charging/discharging time^1–4^. Supercapacitors comprise four major components which are current collectors, active materials, an electrolyte and a separator^5^. Supercapacitors can be categorized as pseudocapacitors and electric double layer capacitors (EDLCs) based on their charge storage mechanism.

Faradaic redox reactions occur at an electroactive material surface in order to store charge in pseudocapacitors. In contrast, the charges are stored electrostatically in EDLCs, where the adsorption/desorption of ions occurs at the electrode/electrolyte interface. Conducting polymers (CPs) and transition metal oxides (TMOs) are classified as pseudocapacitor materials while EDLCs are derived from carbon-based materials such as graphene oxide (GO), multi-walled carbon nanotube (MWCNT), reduced graphene oxide (rGO) and activated carbon. Pseudocapacitors exhibit relatively high specific capacitance than EDLCs, however, it shows low specific power and short cycling life. In comparison to pseudocapacitors, EDLCs show higher specific power, excellent life cycle but lower specific energy. Thus, the combination of pseudocapacitors and EDLCs as hybrid supercapacitors could enhance the supercapacitive performance by exhibiting excellent specific capacitance, superior cyclability, enormous specific power and satisfying specific energy.

Poly(3,4-ethylenedioxythiophene) (PEDOT), polyaniline (PANI), polypyrrole (PPy), polythiophene (PTh) and its derivatives are promising CP candidates for supercapacitor applications. However, PEDOT has received more attention among CPs candidates because of its outstanding properties i.e. good electrical conductivity, wide operating potential, environmentally friendly^6^ and excellent stability at its oxidized state^1,7^. Over the last few years, metal–organic frameworks (MOFs) have received great attention and are labeled as the most promising electrochemical candidate in supercapacitor application due to their high internal pore volume^8^ and high surface area^9^. MOFs are crystalline porous solids, which consist of metal ions as well as organic linkers that are held together by strong covalent bonds^4,10^. HKUST-1 is a copper-based MOF composed of copper ion (metal ion) as well as trimesic acid (organic ligand)^11,12^. HKUST-1 is being explored in supercapacitor application because it exhibits high surface area^13^, superior pore volume^14^, and high thermal stability^1^. However, HKUST-1 suffers from poor electrical conductivity. CPs and MOFs are usually incorporated with carbon-based materials to enhance their properties by providing high mechanical strength and electrical conductivity^15^. One of the carbon-based materials which can boost the stability of CP/MOF composite is rGO. Zhu et al.^16^ reported *p*-toluenesulfonic doped PPy/rGO composite possesses an excellent specific capacitance (280.3 F/g) and the presence of rGO successfully enhanced the stability of PPy with 92% energy retention even after 10,000 cycles. A zinc-based MOF (MOF-5)/rGO composite was prepared by Wen et al*.*^17^ via a solvothermal followed by the annealing process. The composite displayed an enhanced specific capacitance (312 F/g) and 81% retention of capacitance over 5000 cycles. A solid-type symmetrical HKUST-1/rGO deposited on a carbon fiber paper also revealed an excellent charge storage capacity (198 F/g) along with an excellent cyclability^18^.

Here, we successfully fabricated a novel composite consisting of poly(3,4-ethylenedioxythiophene)-reduced graphene oxide/copper-based metal–organic framework (PrGO/HKUST-1) with excellent performance for energy storage. The PrGO was electrochemically prepared while HKUST-1 was synthesized hydrothermally and it was drop casted on top of the PrGO layer. The synthesized PrGO/HKUST-1 was then characterized by FESEM, elemental mapping, XRD, Raman spectroscopy, XPS as well as electrochemical measurements. PEDOT was chosen because of its good electrical conductivity while HKUST-1 possesses a high surface area. A synergistic effect between PEDOT, rGO and HKUST-1 leads to an excellent specific capacitance, specific energy, high specific power and superior cycling life.

## Results and discussion

**Figure 1:**
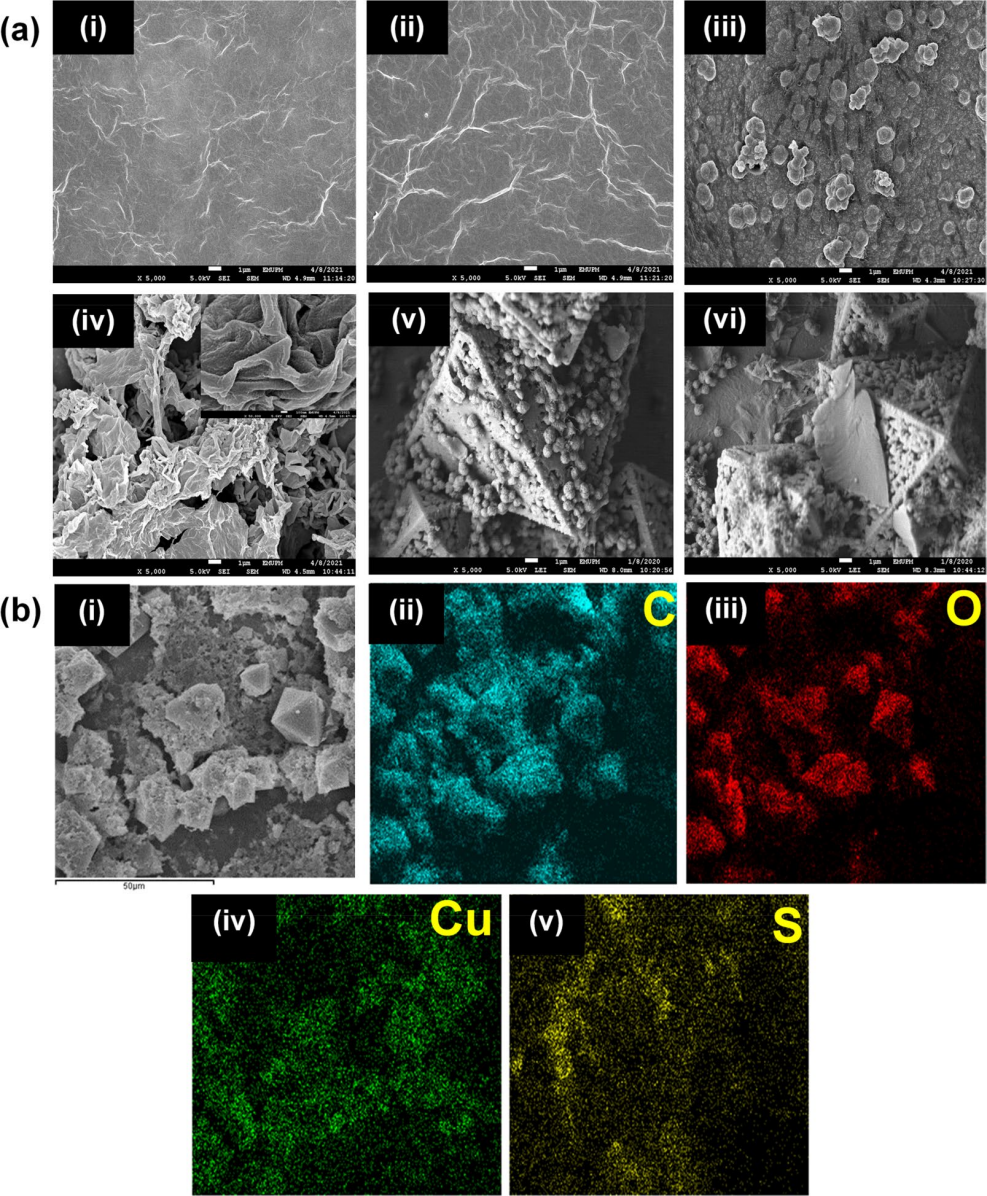
(**a**) FESEM images of (i) GO, (ii) rGO, (iii) PEDOT, (iv) PrGO (inset: higher magnification FESEM image of PrGO), (v) HKUST-1, (vi) PrGO/HKUST-1 and (**b**) elemental mapping of (i) PrGO/HKUST-1 (scale bar: 50 μm) with (ii) C, (iii) O, (iv) Cu and (v) S distributions.

FESEM was performed to identify the surface morphology of the materials as depicted in Fig. 1(a). A wrinkle-like morphology is observed for both GO (Fig. 1(a)(i)) and rGO (Fig. 1(a)(ii)). Comparatively, rGO possesses pronounce wrinkle-like sheet morphology, which is due to the reduction of GO. PEDOT (Fig. 1(a)(iii)) reveals a homogeneous granular morphology similar to a typical polymeric structure. PrGO displays a prominent wrinkle-like sheet morphology (Fig. 1(a)(iv)), which is contributed by the rGO. The granular PEDOT that is covered on the rGO sheet demonstrates the successful incorporation of PEDOT and rGO (inset of Fig. 1(a)(iv)). This wrinkled-like sheet morphology provides a high surface area which enables an efficient ion diffusion. The HKUST-1 (Fig. 1(a)(v)) displays a typical octahedral shape morphology, whereas the PrGO/HKUST-1 demonstrates the presence of HKUST-1 and PrGO as both the octahedral morphology as well as wrinkle-like rGO sheet covered with PEDOT grains are observed in Fig. 1 (a)(vi).

The PrGO/HKUST-1 (Fig. 1(b)(i)) was further analyzed via elemental mapping (Fig. 1(b)(ii-v)) to study the elements that exist in the composite. Carbon (C), oxygen (O), sulfur (S) and copper (Cu) are evenly distributed on the PrGO/HKUST-1 surface, indicating a homogeneous formation of the composite. S and Cu elements signify the distribution of PEDOT and HKUST-1, respectively^19,20^. The presence of C and O elements are originated from PEDOT, rGO and HKUST-1.

**Figure 2:**
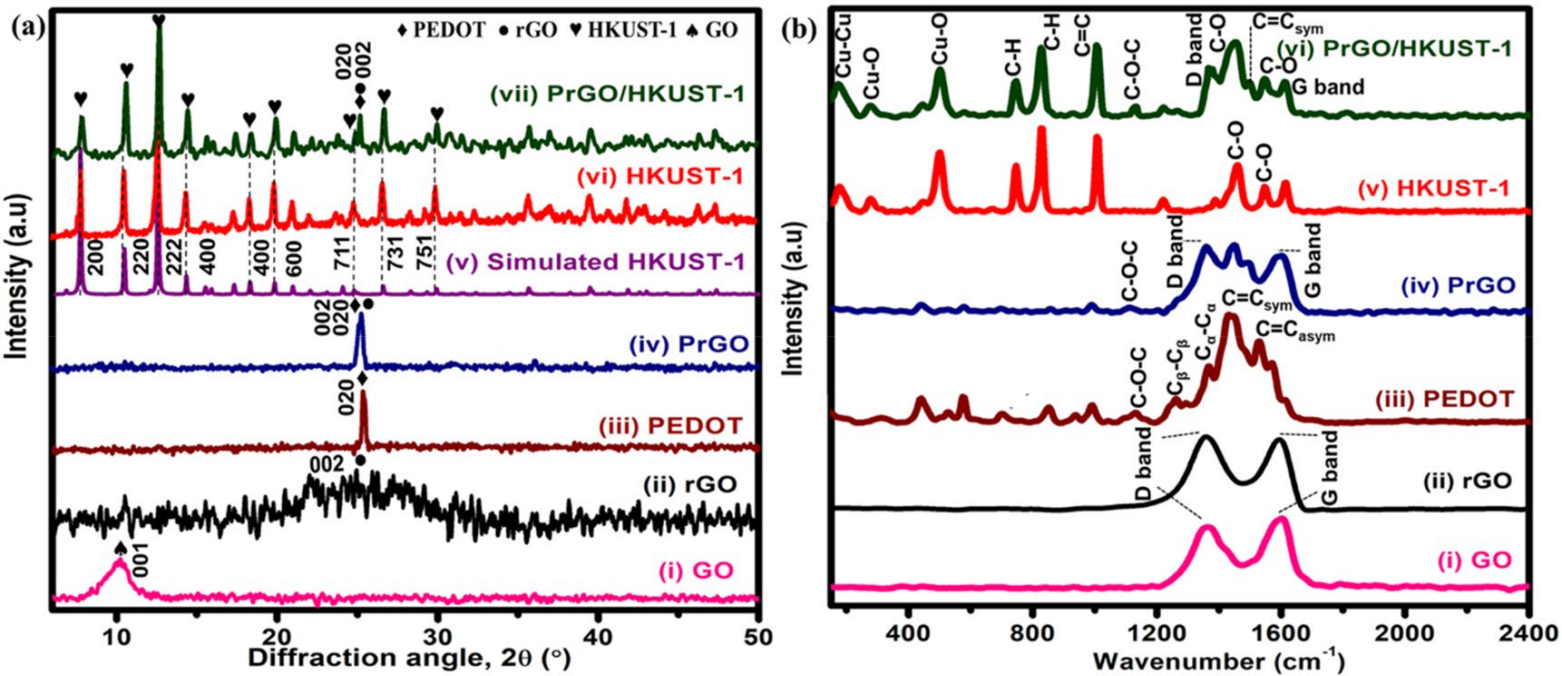
(**a**) XRD patterns and (**b**) Raman spectra of GO, rGO, PEDOT, PrGO, HKUST-1 and PrGO/HKUST-1.

The crystallinity of the composites was evaluated using XRD (Fig. 2(a)) analysis. The peak at 2θ = 10.1° (Fig. 2(a)(i)) represents lattice plane (001) of GO. The XRD spectrum of rGO (Fig. 2(a)(ii)) implies a broad peak at 2θ = 25.0° (002), which confirm the successful reduction of GO to rGO^21,22^ whereas Fig. 2(a)(iii) implies a diffraction peak at 2θ = 25.7° (020), verifying the presence of interchain planar ring stacking of PEDOT^23,24^. PrGO (Fig. 2(a)(iv)) composite reveals a diffraction peak (2θ = 25.6°), representing the (020) and (002) lattice planes of PEDOT and rGO, respectively. The XRD peak of PrGO only shows one diffraction peak (2θ = 25.6°) as the peak of rGO is overlapping with PEDOT. The disorder in the rGO sheets appears when the majority of oxygenated functional groups was successfully reduced from the GO sheet during the electrodeposition^25^. The PrGO composite does not show any diffraction peak at 2θ = 10° (peak for GO), which further verifies the reduction of GO to rGO^26^. The as-synthesized HKUST-1 (Fig. 2(a)(vi)) displays similar diffraction peaks as the simulated HKUST-1 (Fig. 2(a)(v)) at 6.7° (200), 9.5° (220), 11.6° (222), 13.5° (400), 17.5° (400), 19.1° (600) and 29.4° (751), revealing a successful synthesis of HKUST-1 via hydrothermal^27,28^. All the XRD diffraction peaks of PEDOT, rGO and HKUST-1 are well-presented in the PrGO/HKUST-1 (Fig. 2(a)(vii)) spectrum. The results demonstrate that the framework of HKUST-1 is retained during the synthesis process which is well supported by the FESEM images (Fig. 1(a)(v-vi).

The vibrational modes of different materials were examined via Raman spectroscopy (Fig. 2(b)). GO (Fig. 2(b)(i)) and rGO (Fig. 2(b)(ii)) imply two intense Raman peaks at 1360 and 1598 cm^−1^, resembling the D band (*sp*^3^-hybridized carbon) and G band (*sp*^2^-hybridized carbon), respectively. The intensity ratio of the D and G bands (I_D_/I_G_) represents the degree of disorder in graphitic material^29^. The calculated I_D_/I_G_ ratio of GO and rGO are 0.9 and 1.2, respectively. The I_D_/I_G_ ratio of more than 1 indicates the presence of high *sp*^3^-hybridized carbon atoms compared to *sp*^2^-hybridized carbon^30^. The obtained I_D_/I_G_ ratio of rGO (1.2) depicts the successful reduction of GO to rGO. PEDOT (Fig. 2(b)(iii)) displays peaks at 440, 576, and 989 cm^−1^, corresponding to the oxyethylene ring of EDOT monomer deformation. The C–O–C mode can be observed at 1107 cm^−1^ while C_β_-C_β_, C_α_-C_α_, symmetry and asymmetry C = C stretching modes of PEDOT are seen at 1260, 1366, 1427 and 1526 cm^−1^, respectively^7^. The PrGO (Fig. 2(b)(iv)) also displays two obvious peaks of rGO at 1356 (D band) and 1600 cm^−1^ (G band) and the calculated I_D_/I_G_ ratio of PrGO (1.2) demonstrates a high degree of disorder in PrGO, revealing the majority of oxygenated functional groups in GO have been reduced successfully^31^. HKUST-1 (Fig. 2(b)(v)) displays all vibration modes of Cu(II) species at the low frequency region (150 to 600 cm^−1^). The Raman peak at 177 cm^−1^ exhibits the presence of Cu-Cu dimer stretching mode, while the Cu–O vibration modes of HKUST-1 can be detected at 278 and 501 cm^−1^. The C-H out-of-plane ring bending modes of trimesic acid are observed at 744 and 827 cm^−1^ whereas the C=C stretching mode of the trimesic acid benzene ring is spotted at 1006 cm^−1^. Raman peaks of HKUST-1 (Fig. 2(b)(v)) are observed at 1457 and 1544 cm^−1^, indicating the asymmetry and symmetry C-O stretching modes, respectively. HKUST-1 incorporated with PrGO exhibits all Raman peaks of PEDOT, rGO and HKUST-1, demonstrating the successful combination of PrGO/ HKUST-1 (Fig. 2(b)(vi)).

**Figure 3:**
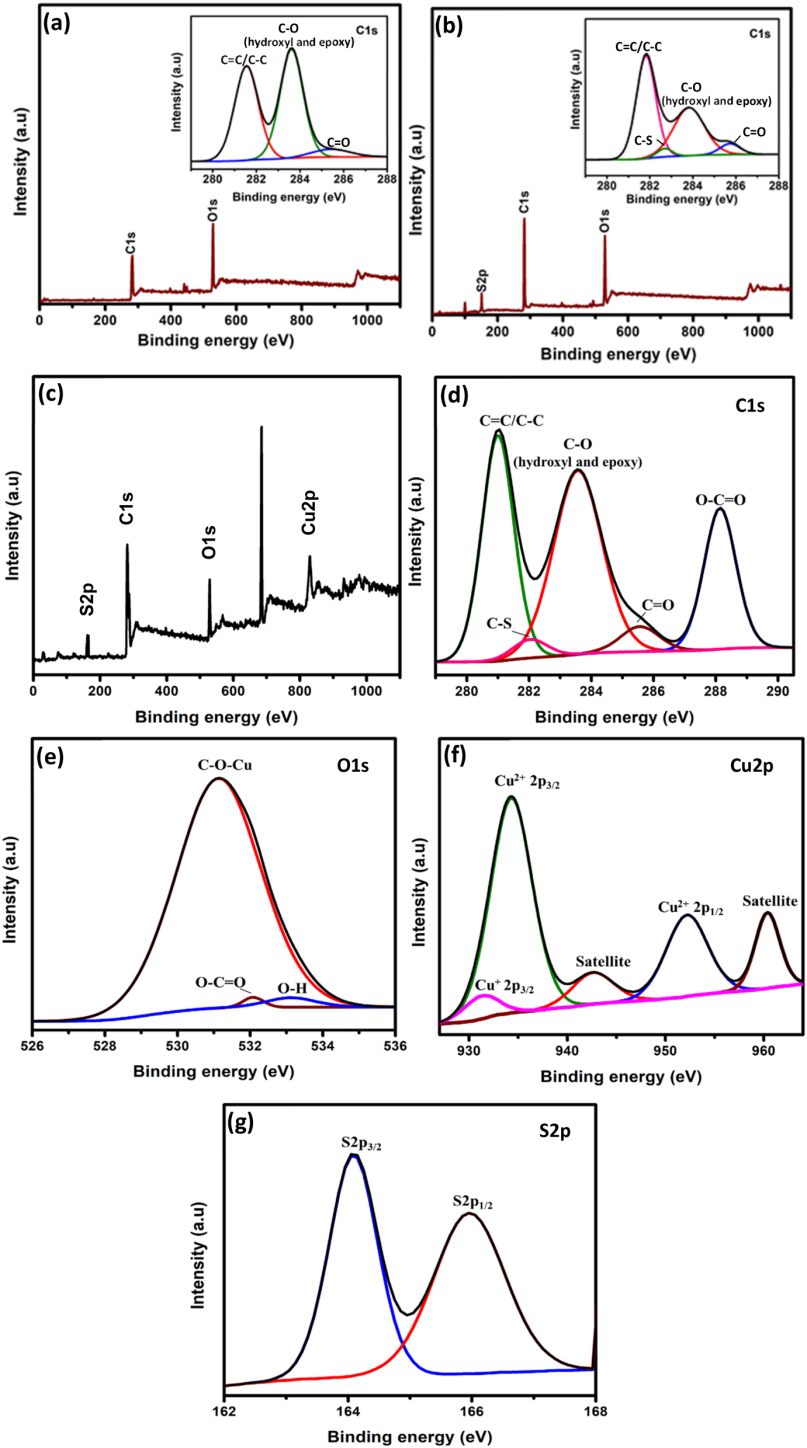
Wide scan XPS spectra of (**a**) GO (inset: high resolution C1s), (**b**) PrGO (inset: high resolution C1s) and (**c**) PrGO/HKUST-1. High resolution (**d**) C1s, (**e**) O1s, (**f**) Cu2p and (**g**) S2p of PrGO/HKUST-1.

XPS analysis was performed in order to determine the chemical composition of the as-prepared samples. The C1s and O1s signals are observed in GO (Fig. 3(a)) while PrGO (Fig. 3(b)) implies the S2p, C1s and O1s signals. The inset of Fig. 3(a) signifies three main C1s peaks of GO, which appear at 281.5 eV (C=C/C–C), 283.6 eV (C–O (epoxy and hydroxy)) and 285.4 eV (C=O)^32^. The C=C/C–C, C–O (epoxy and hydroxy), C=O and C–S interactions in PrGO are identified at 281.8, 283.8, 285.7 and 282.6 eV, respectively. The intensity of C-O (epoxy and hydroxy) in PrGO is comparatively lower than the GO. PrGO also possesses a higher C/O ratio of 1.4 compared to GO (1.0), manifesting removal of a large fraction of oxygenated functional groups within the GO sheet as a result of the electrochemical reduction reaction^33,34^. The wide scan XPS spectrum of PrGO/HKUST-1 (Fig. 3(c))) indicates the existence of sulfur (S), carbon (C), oxygen (O) and copper (Cu) in the PrGO/HKUST-1 composite. The high-resolution C1s XPS spectrum (Fig. 3(d)) denotes five peaks, which appear at 281, 282, 283.6, 285.5, and 288.1 eV, implying the C=C/C–C, C–S, C–O (epoxy and hydroxy), C=O and O–C=O in the composite^7,35^. The O1s spectrum (Fig. 3(e)) is deconvoluted into three peaks, verifying the C-O-Cu (531.2 eV), O-C = O (532.1 eV) and O–H (533.0 eV) interactions. The Cu2p (Fig. 3(f)) of HKUST-1 depicts Cu^+^2p_3/2_, Cu^2+^2p_3/2_ and Cu^2+^2p_1/2_ peaks at 931.5, 934.3 and 952.4 eV, respectively. The binding energies of 942.8 and 960.4 eV are the satellite peaks that imply the characteristics of HKUST-1^36^. Figure 3(g) with S2p_3/2_ (164.0 eV) and S2p_1/2_ (165.9 eV) peaks affirm the presence of PEDOT^6^.

**Figure 4:**
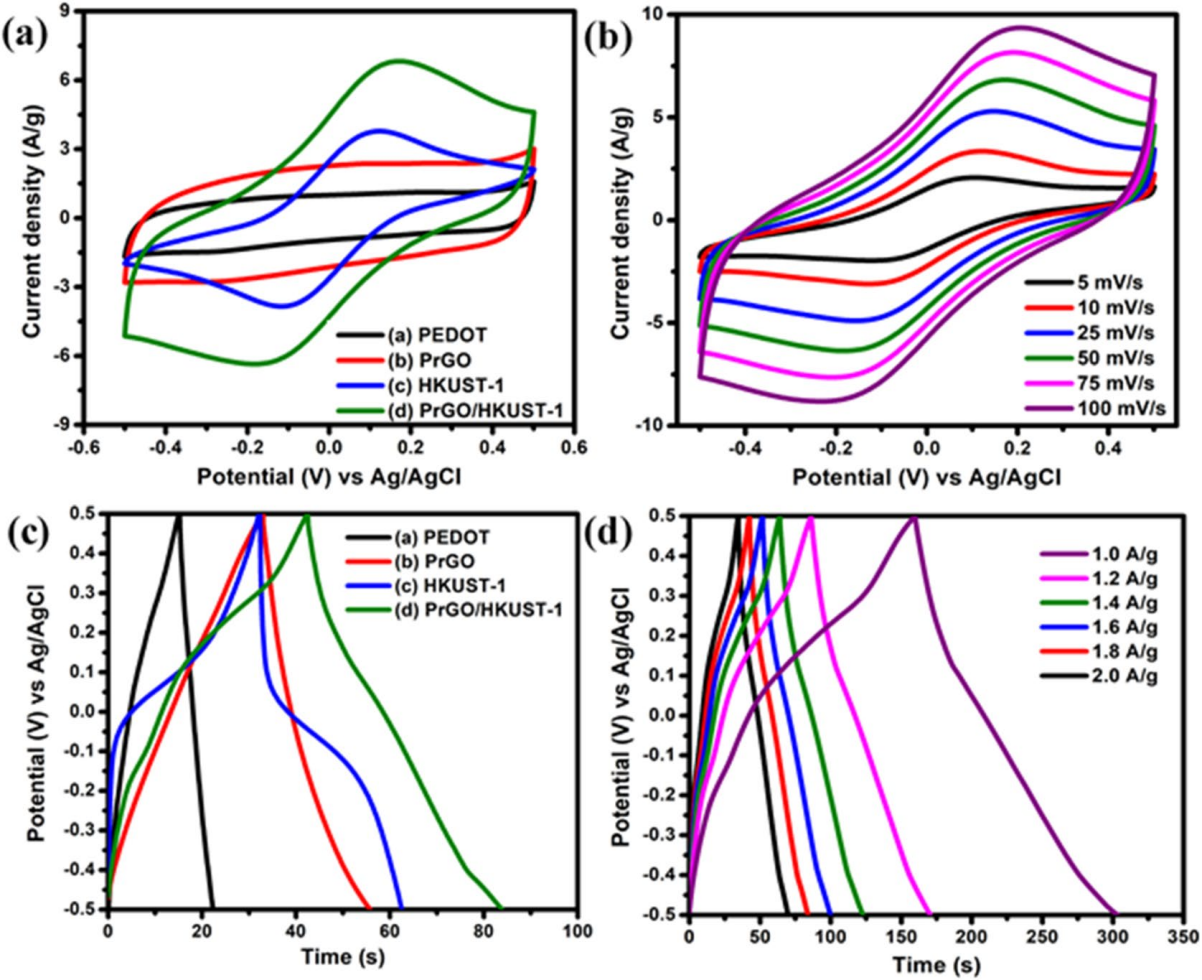
(**a**) CV curves of PEDOT, PrGO, HKUST-1 and PrGO/HKUST-1 at 50 mV/s, (**b**) CV curves of PrGO/HKUST-1 at various scan rates (5–100 mV/s), (**c**) GCD analysis of PEDOT, PrGO, HKUST-1, PrGO/HKUST-1 and (**d**) GCD plots of PrGO/HKUST-1 at various current density (1.0–2.0 A/g).

The electrochemical properties of the composites were evaluated in a three-electrode configuration. From Fig. 4(a), PEDOT reveals a quasi-rectangular CV shape, suggesting the pseudocapacitance characteristic. PrGO displays a nearly rectangular CV curve, indicating EDLC characteristics. This result shows that rGO is dominant in the PrGO composite. CV curve of HKUST-1 displays a redox peak that confirms the faradic charge storage mechanism. Interestingly, the integration of PrGO with HKUST-1 (PrGO/HKUST-1) has significantly increased the redox peak currents, where the peaks are mainly contributed by the pseudocapacitance characteristic of HKUST-1. The oxidation peak demonstrates the oxidation of Cu^+^ to Cu^2+^ while, the reduction peak shows the reduction of Cu^2+^ to Cu^+^^37^. The electrochemical reactions that occur in the HKUST-1 can be explained using Eq. (1)^38^:

1 $$({\text{Cu}}^{2 + } {\text{R}})_{{\text{n}}} + {\text{nK}}^{ + }_{{({\text{gel electrolyte}})}} + {\text{ne}}^{ - } \rightleftharpoons \left( {{\text{Cu}}^{ + } ({\text{K}}^{ + } ){\text{R}}} \right)_{{\text{n}}}$$

where R is the ligand of HKUST-1. When electrolyte cation (K^+^) enters the HKUST-1 network, HKUST-1 displays pseudocapacitive behavior as a reaction between copper ion (Cu^2+/+^) and the electrolyte occurs, which mainly contributed from the K^+^ ion insertion and deinsertion process^38^. Figure 4(b) implies the CV curves of PrGO/HKUST-1 at different scan rates ranging from 5 to 100 mV/s. The redox current density of PrGO/HKUST-1 gradually intensifies as the scan rate increases. It can be clearly observed that PrGO/HKUST-1 is able to maintain its CV shape with well-defined redox peaks without an evident distortion at a higher scan rate (100 mV/s), signifying a good rate capability of PrGO/HKUST-1^39,40^.

The prepared materials were further analyzed via galvanostatic charge–discharge (GCD) analysis. Figure 4(c) illustrates the GCD curves of different materials at 1.8 A/g. PEDOT, HKUST-1 as well as PrGO/HKUST-1 demonstrate non-linear GCD curves^41^, indicating the good capacitive performance of materials with pseudocapacitive behavior^11,40,42^ whereas PrGO exhibits a nearly linear GCD curve, indicating EDLC behavior of the electrode^18^. PrGO/HKUST-1 depicts the longest discharging time compared to other individual samples with a small and negligible voltage drop (*IR* drop), demonstrating an outstanding specific capacitance as well as the low internal resistance of the electroactive material^43^. Figure 4(d) presents GCD measurements of PrGO/HKUST-1 at different current densities (1.0—2.0 A/g). The GCD curves of PrGO/HKUST-1 clearly show the discharging time of the electrode reduces when the current density increases. This is because, at higher current density, the electrolyte ions movement is time limited, where only outer electroactive sites of the electrode are involved for the energy storage process. Moreover, the GCD curves retain non-linear GCD shapes at all current densities, demonstrating good electrochemical reversibility of PrGO/HKUST-1^44^.

**Figure 5:**
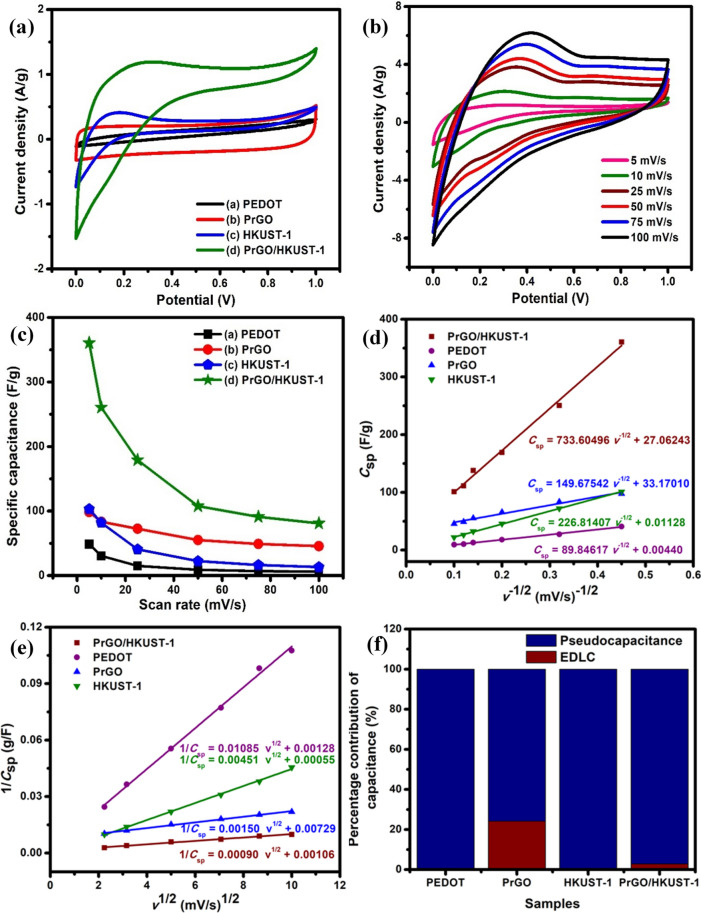
(**a**) CVs of PrGO, PEDOT, HKUST-1 and PrGO/HKUST-1 using KCl/PVA gel electrolyte at 5 mV/s, (**b**) CVs of symmetrical PrGO/HKUST-1 at different scan rates (5–100 mV/s). The graph of (**c**) specific capacitance versus scan rate, Trasatti plots of (**d**) *C*_sp_ against 1/square root of scan rate (v^−1/2^), (e) 1/*C*_sp_ against *v*^1/2^ and (**f**) percentage contribution of capacitance for PrGO, PEDOT, HKUST-1 and PrGO/HKUST-1.

The charge storage capacity of the as-prepared symmetrical energy storage devices was examined via a two-electrode configuration using KCl/PVA gel as an electrolyte and separator^1^. Figure 5(a) depicts the CV curves of different materials at a potential range of 0 to 1 V. Quasi rectangular CV shapes of PEDOT and HKUST-1 prove the pseudocapacitance behavior, whereas PrGO displays a nearly rectangular CV curve, demonstrating EDLC characteristic. The PrGO/HKUST-1 depicts a quasi rectangular CV curve, suggesting a combination of EDLC and pseudocapacitance behavior^45^. The PrGO/HKUST-1 reveals the largest CV curve, signifying the highest specific capacitance (*C*_sp_) as the area under the CV curve indicates the quantity of electrical charge stored in an electrode^13^. The *C*_sp_ can be calculated using Eq. (2):

2 $$C_{sp} = \frac{1}{2mv\Delta V}\int {IdV}$$

where *I*d*V* indicates the integrated area of the CV curve while *m*, *v* and ∆V are mass (g) of active material, potential scan rate (V/s) and potential window (V) of the sample, respectively. The *C*_sp_ obtained for PrGO/HKUST-1 is 360.5 F/g where it is significantly greater compared to HKUST-1 (103.1 F/g), PrGO (98.5 F/g) and PEDOT (50.8 F/g) at a scan rate of 5 mV/s. Figure 5(b) implies the CV curves of symmetrical PrGO/HKUST-1 from the scan rate of 5 to 100 mV/s. The current density and the area under the CV curves increase evidently as the scan rate is increased. The relationship between *C*_sp_ and scan rate is elucidated in Fig. 5(c) and the results confirm that the *C*_sp_ reduces over the increasing scan rate. At slower scan rates, the electrolyte ions are able to utilize all the electroactive sites of the material and lead to a complete redox reaction, which provides high *C*_sp_^4^. However, at faster scan rates, the movement of electrolyte ions is time limited which means that only the outer electroactive sites of the material are involved for energy storage, resulting in low *C*_sp_^46^. Interestingly, PrGO/HKUST-1 exhibits higher *C*_sp_ compared to other materials, proving incorporation of HKUST-1, PEDOT and rGO can successfully boost the electrochemical performance of PrGO/HKUST-1 composite, which mainly caused by the faradic redox reaction that occurs at the surface of electroactive material^47^. The *C*_sp_ achieved in this work is higher in comparison to other reported HKUST-1 based supercapacitors (Table 1).

**Table 1:** Comparison of specific capacitance of proposed symmetric supercapacitor with other reported HKUST-1 based energy storage devices.

Material	*C*_sp_ (F/g) in two-electrode configuration	Electrolyte	References
rGO/HKUST-1	193.0 at 5 mV/s	NaNO_3_/PVA	^18^
PEDOT:PSS@HKUST-1	47.2 at 10 mV/s	1 M NaNO_3_	^48^
Cu-MOF derived Cu–C	8.3 at 0.4 A/g	1 M KOH	^49^
HKUST-1/PANI	19.9 at 0.5 A/g	6 M KOH	^50^
PrGO/HKUST-1	360.5 at 5 mV/s	KCl/PVA	This work

rGO/HKUST-1: reduced graphene oxide/HKUST-1, PEDOT:PSS@HKUST-1: poly(3,4-ethylenedioxythiophene:polystyrene sulfonate@HKUST-1, Cu-MOF derived Cu-C: copper-carbon derived from HKUST-1 and HKUST-1/PANI: HKUST-1/polyaniline.

Trasatti method was performed to evaluate the individual capacitance contribution (EDLC and pseudocapacitance) from the total capacitance (*C*_t_)^51^. The EDLC (surface charge) capacitance (C_EDLC_) is achieved by retrieving the y-axis intercept of plot *C*_sp_ vs 1/square root of scan rate (*v*^−1/2^) (Eq. (3)). Whereas, Eq. (4) is used to determine the *C*_t_ by extracting the 1/C_t_ value (y-axis intercept of plot 1/*C*_sp_ vs square root of scan rate (*v*^1/2^)). The capacitance difference between *C*_t_ and *C*_EDLC_ is expressed as the diffusion-controlled charge (pseudocapacitance, *C*_PC_)^52^. Figure 5(d) denotes the relationship between *C*_sp_ and *v*^−1/2^. PrGO and PrGO/HKUST-1 illustrate C_EDLC_ of 33.2 F/g and 27.1 F/g, respectively. Meanwhile, PEDOT and HKUST-1 depict minimal C_EDLC_, designating the pseudocapacitive behavior of the samples. The *C*_t_ values of the as-prepared samples are retrieved from Fig. 5(e). The percentage of *C*_EDLC_ (%*C*_EDLC_) and *C*_PC_ (%*C*_PC_) contribution in the samples are calculated utilizing Eqs. (5) and (6), respectively. The presented bar chart (Fig. 5(f)) illustrates a maximum %*C*_PC_ of 99.9% for both PEDOT and HKUST-1, indicating the pseudocapacitive charge storage mechanism. Whereas, the %*C*_EDLC_ and %*C*_PC_ in PrGO (%*C*_EDLC_ = 24.2% and %*C*_PC_ = 75.8%) and PrGO/HKUST-1 (%*C*_EDLC_ = 2.9% and %*C*_PC_ = 98.1%) demonstrate hybrid supercapacitive behavior with both surface charge and diffusion-controlled charge.

3 $$C_{{{\text{sp}}}} = constant \cdot v^{{ - \frac{1}{2}}} + C_{{{\text{EDLC}}}}$$

4 $$\frac{1}{{C_{{{\text{sp}}}} }} = constant \cdot v^{\frac{1}{2}} + C_{{\text{t}}}$$

5 $$\% C_{{{\text{EDLC}}}} = \frac{{C_{{{\text{EDLC}}}} }}{{C_{{\text{t}}} }} \times 100$$

6 $$\% C_{{{\text{PC}}}} = \frac{{(C_{t} - C_{{{\text{EDLC}}}} )}}{{C_{{\text{t}}} }} \times 100$$

**Figure 6:**
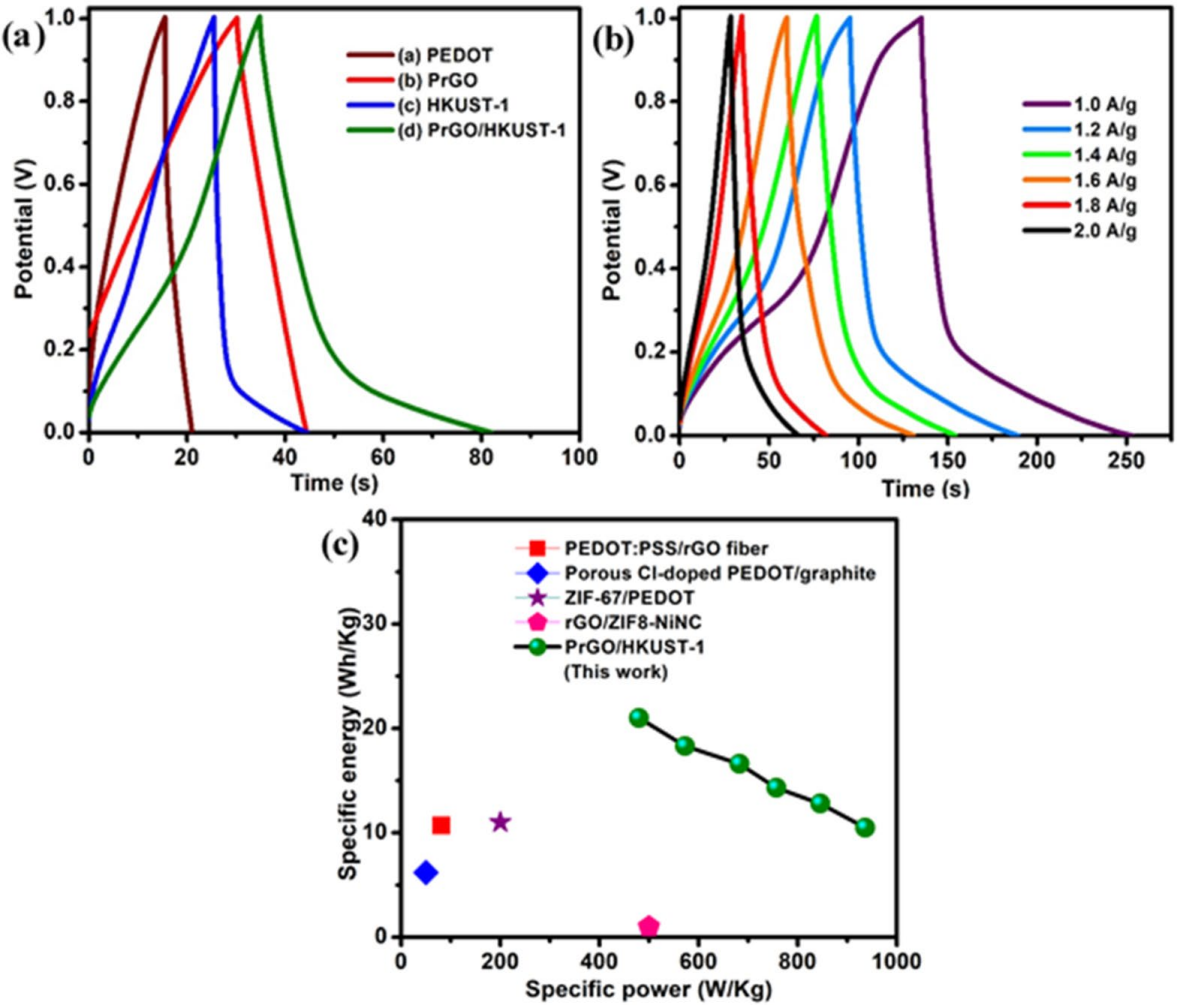
(**a**) GCD analysis of PEDOT, PrGO, HKUST-1 and PrGO/HKUST-1 at a current density of 1.8 A/g using KCl/PVA gel electrolyte, (**b**) GCD analysis of symmetrical PrGO/HKUST-1 at various current densities (1.0–2.0 A/g), (**c**) Ragone plots of PrGO/HKUST-1.

The as-prepared symmetrical devices were further compared to other devices via GCD measurements at a current density of 1.8 A/g. The symmetrical PrGO/HKUST-1 (Fig. 6(a)) device exhibits a non-linear GCD curve, revealing the presence of pseudocapacitive material that is dominant in the PrGO/HKUST-1. Furthermore, the symmetrical PrGO/HKUST-1 shows the longest discharging time, suggesting a high *C*_sp_. Figure 6(b) displays GCD measurements of symmetrical PrGO/HKUST-1 device at various current densities (1.0 to 2.0 A/g). The *C*_sp_ can be also obtained from GCD measurements utilizing Eq. (7), where the *I*, ∆t, *m* and ∆V refer to discharging current (A), discharging time of the device (s), average mass of two electrodes (g) and cell operating potential (V), respectively.

7 $$C_{{{\text{sp}}}} = \frac{I\Delta t}{{m\Delta V}}$$

The PrGO/HKUST-1 device exhibits a *C*_sp_ of 163.5 F/g at 1.0 A/g, which declines to 104.2 F/g at 2.0 A/g. The GCD results (Fig. 6(b)) are in good agreement with the CV plots displayed in Fig. 5(b). The specific energy (*E*) and specific power (*P*) of an electrode can be measured utilizing Eqs. (8) and (9) where *C*_sp_, ∆V, *I* and *m* are the specific capacitance, potential window at discharging process, the current applied and mass of symmetrical electrode, respectively.

8 $$E = \frac{{C_{{{\text{sp}}}} \Delta V^{2} }}{2}$$

9 $$P = \frac{\Delta VI}{{2m}}$$

Figure 6(c) reveals specific energy of 21 Wh/kg for PrGO/HKUST-1 at a specific power of 479.7 W/kg, where it is significantly greater compared to recently reported symmetrical supercapacitors (Table 2).

**Table 2:** Comparison of specific energy and specific power of proposed symmetric supercapacitor with other reported symmetrical supercapacitors.

Material	Specific energy (Wh/kg)	Specific power (W/kg)	References
PEDOT:PSS/rGO fiber	10.7	81.3	53
porous Cl-doped PEDOT/graphite	6.2	50.1	54
ZIF-67/PEDOT	11.0	200.0	55
rGO/ZIF8-NiNC	~ 1.0	500.0	56
PrGO/HKUST-1	21.0	479.7	This work

PEDOT:PSS/rGO fiber: poly(3,4-ethylenedioxythiophene):poly(styrenesulfonic acid)/reduced graphene oxide, porous Cl-doped PEDOT/graphite: chloride ions doped poly (3, 4-ethylenedioxythiophene) porous nanostructures on graphite, ZIF-67/PEDOT: zeolitic imidazole frameworks-67/ poly(3,4-ethylenedioxythiophene), rGO/ZIF8-NiNC: reduced graphene oxide/zeolitic imidazole framework-8 nickel nanocone.

**Figure 7:**
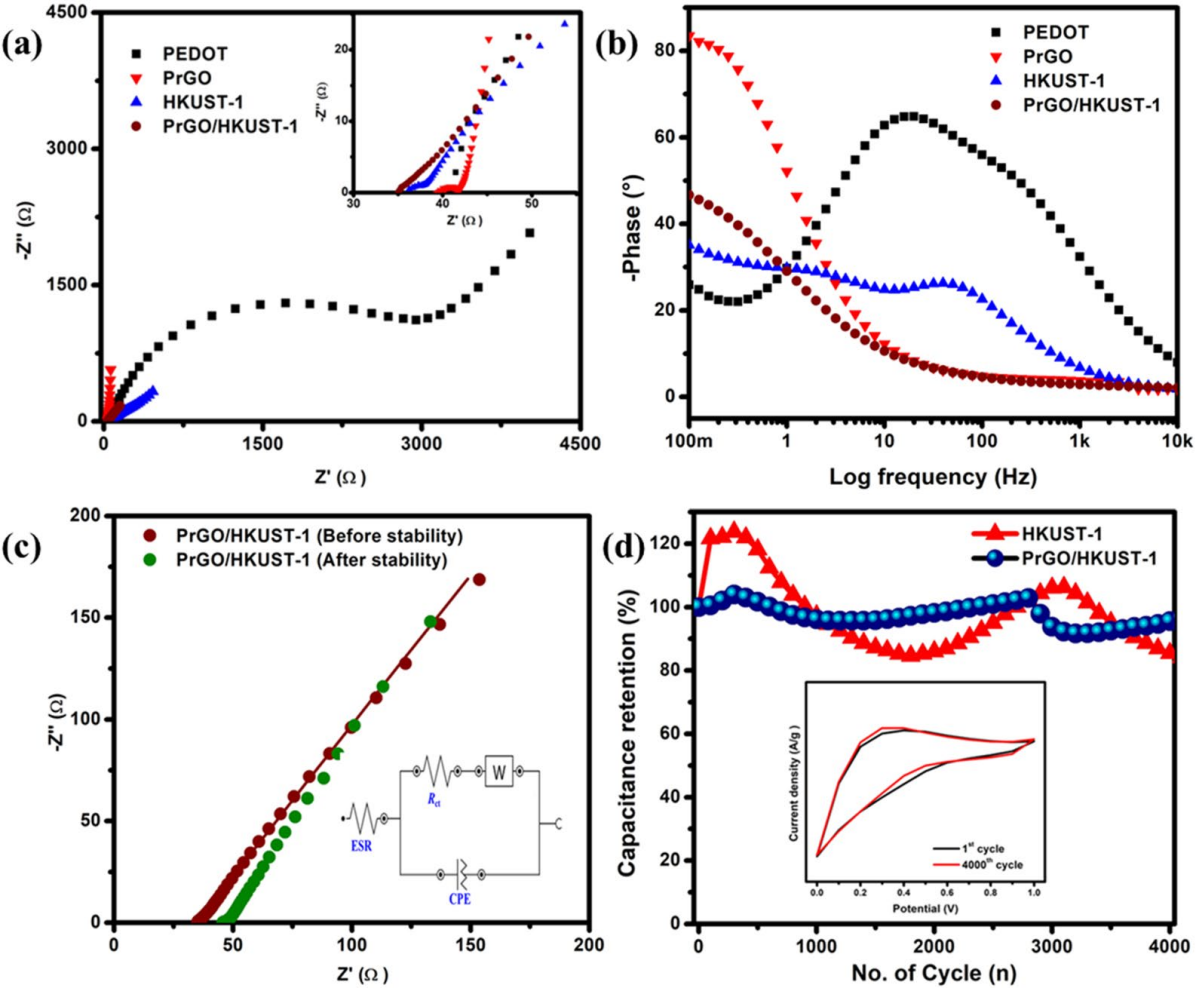
(**a**) Nyquist and (**b**) Bode plots of PrGO, PEDOT, HKUST-1 and PrGO/HKUST-1 between 0.1 Hz and 10 kHz (inset displays at high-frequency region). (**c**) Nyquist plots of PrGO/HKUST-1 (before and after stability) with an equivalent circuit and (**d**) cycling stability over 4000 cycles at 100 mV/s (Inset: CV curves of PrGO/HKUST-1 at 1^st^ and 4000^th^ cycle).

The conductivity as well as the ion mobility at the interface of electrode/electrolyte were evaluated via EIS analysis by retrieving the information of the internal resistance along with the interface resistance amidst an electrode and electrolyte^57^. The Nyquist plots (Fig. 7(a)) consist of equivalent series resistance (ESR) as well as the resistance of charge transfer (*R*_ct_) at the high frequency region, while the vertical line (Warburg line) at low frequency region. The ESR is the intersection point that appears at the real axis whereas *R*_ct_ is the semicircle diameter. The PrGO/HKUST-1 exhibits the lowest ESR (35.0 Ω) and *R*_ct_ (1.16 Ω) compared to PEDOT (ESR = 40.1 Ω, *R*_ct_ = 3.7 kΩ), PrGO (ESR = 39.3 Ω, *R*_ct_ = 2.72 Ω) and HKUST-1 (ESR = 36.2 Ω, *R*_ct_ = 2.56 Ω). The lowest ESR of PrGO/HKUST-1 reveals a good contact between the current collector and electrode material while the small *R*_ct_ shows a low resistance at the electrode/electrolyte interface, demonstrating high conductivity of PrGO/HKUST-1^57^. Moreover, PrGO/HKUST-1 illustrates the shortest vertical line at the low-frequency region, signifying a rapid ion diffusion rate within the electrode/electrolyte interface^58^.

Bode plots (Fig. 7(b)) illustrate the phase angle of the as-prepared samples with respect to the applied frequency. The phase angle of PEDOT, HKUST-1 and PrGO/HKUST-1 are 25.9°, 35.1° and 46.7°, respectively. The lower phase angle affirms the pseudocapacitive nature of PEDOT, HKUST-1 and PrGO/HKUST-1^59^. Meanwhile, the phase angle of PrGO (83.5°) approaches 90°, signifying ideal capacitive behavior^60^. These results are in good agreement with the Nyquist plots (Fig. 7(a)) and GCD analyses (Fig. 4(c)).

Inset Fig. 7(c) displays an equivalent circuit that represents the electrochemical system of the PrGO/HKUST-1 composite. The equivalent circuit consists of ESR, *R*_ct_, Warburg (W) and the constant phase element (CPE). The double layer capacitor (*C*_dl_) is replaced by CPE due to the electrode surface inhomogeneity^7^. Chi-square (χ^2^) is the sum of the square differences between theoretical and experimental results^61^. From the fit and simulation analysis, the value of χ^2^ obtained is 7.2 × 10^–3^, proving that the equivalent circuit is suitable for the electrochemical system of PrGO/HKUST-1.

The cycling stabilities of HKUST-1 and PrGO/HKUST-1 were evaluated over 4000 CV cycles at 100 mV/s. From Fig. 7(d), the capacitance retention of PrGO/HKUST-1 is 95.5% compared to HKUST-1 (85.4%), confirming excellent long-term stability of the PrGO/HKUST-1 device. A slight increment in capacitance retention (first 300 cycles) can be noticed in both HKUST-1 (121.3%) and PrGO/HKUST-1 (103.2%), indicating a self-activation process where electrolyte ions continuously penetrate all the active sites of the composite^62^. During the long-term cycling stability, HKUST-1 depicts an obvious decrease in specific capacitance compared to PrGO/HKUST-1 due to the swelling and shrinking properties of HKUST-1 during the redox reaction^63^. The inset of Fig. 7(d) demonstrates that the shape and the size of the CV curve at the 4000^th^ cycle are almost similar to the 1st cycle, proving that the specific capacitance of PrGO/HKUST-1 only drops slightly. The high cycling stability of PrGO/HKUST-1 is due to the presence of rGO, where it is able to provide high mechanical strength to the composite^64^. After 4000 cycles, the EIS measurement of the PrGO/HKUST-1 device implies an ESR and *R*_ct_ of 45.6 Ω and 1.19 Ω, respectively (Fig. 7(c)). The almost similar *R*_ct_ of PrGO/HKUST-1 before (1.16 Ω) and after (1.19 Ω) cycling stability test indicates good intrinsic conductivity^65^. The ESR of PrGO/HKUST-1 increases from 35.0 to 45.6 Ω, denoting a slight increment in the ionic resistance of electrolyte^6,66^ and causing a small drop in the capacitance retention (95.5%) in the device.

## Conclusions

A novel PrGO/HKUST-1 composite was successfully synthesized as an outstanding supercapacitor device. The octahedral HKUST-1 on wrinkled-like sheet PrGO exhibited a unique morphology which boosts the electrochemical performance of the electrode by demonstrating a superior specific capacitance (360.5 F/g), remarkable specific energy (21 Wh/kg) at a specific power of 479.7 W/kg and excellent cyclability (95.5% energy retention over 4000 cycles). Thus, the combination of PrGO and HKUST-1 with enhanced electrochemical performance is a promising energy storage material.

## Experimental

### Materials

Indium tin oxide (ITO) glasses (7 Ω) were supplied by Xin Yan Technology Ltd. Acetone (99%) and ethanol (95%) were received from QRec and J.Kollin Chemical, respectively. GO was procured from Graphenea. Copper (II) nitrate trihydrate was acquired from Fisher Scientific while trimesic acid (BTC) was obtained from Sigma-Aldrich. Polyvinyl alcohol (PVA), N-methyl-2-pyrrolidone (NMP), 3,4-ethylenedioxythiophene (EDOT), carbon black, poly(vinylidene fluoride) (PVDF) were received from Sigma-Aldrich. Potassium chloride (KCl) was supplied by Fisher Scientific. Deionized (DI) water (18.5 MΩ cm) from the Merck Millipore-Q was utilized in all experiments.

### Preparation of PrGO/HKUST-1

An aqueous GO solution (1 mg/ml) was initially sonicated for 1 h. 1 mM EDOT monomer was then added into the 1 mg/ml GO and the solution was shaken and left overnight. The PrGO was electrodeposited on ITO glass (current collector) at a fixed potential of 1.2 V for 10 min^7^. For comparison, PEDOT was also prepared via a similar deposition technique using 10 mM EDOT and 0.1 M LiClO_4_ in DI water. The electrodepositions of PEDOT and PrGO were carried out using a potentiostat (Autolab PGSTAT204) in a three-electrode configuration where Ag/AgCl, ITO glass and platinum wire (Pt) were utilized as the reference electrode, working electrode and counter electrode, respectively.

Firstly, 0.88 g copper (II) nitrate trihydrate (Cu(NO_3_)_2_.3H_2_O) was dissolved in 12 mL DI water, while 0.42 g trimesic acid (BTC) was dissolved in 12 mL ethanol. The prepared mixtures were then mixed followed by stirring for 1 h. The resultant mixture was poured into a Teflon-lined autoclave, sealed and heated at 120 °C for 16 h. Finally, HKUST-1 was obtained and dried. A uniform HKUST-1 slurry was obtained by mixing HKUST-1, polyvinylidene fluoride (PVDF) (in N-methylpyrrolidone (NMP)), carbon black at 8:1:1 mass ratio^58^. HKUST-1 slurry (10 μL) was drop coated on the PrGO layer (1 cm^2^) using a micropipette^67^ to obtain PrGO/HKUST-1 composite. The PrGO/HKUST-1 was allowed to dry before proceeding with the electrochemical measurements.

### Fabrication of symmetrical supercapacitor

A KCl/PVA gel was obtained by mixing 1 g PVA as well as 0.75 g KCl in 10 ml DI water, followed by vigorous stirring and heating (~ 90 °C) until a clear and transparent gel was obtained^68^. Two identical PrGO/HKUST-1 electrodes (1 cm^2^) were then sandwiched together, separated by KCl/PVA gel electrolyte. The KCl/PVA gel acted as an electrolyte and an ion-porous separator.

### Material characterizations

The phase identification was analyzed using X-Ray diffraction (XRD) (Shimadzu with Cu Kα radiation (λ = 1.54 Å). Raman spectroscopy (Alpha300 R microscopic confocal Raman spectrometer (WITec GmbH), 532 nm) was used to examine the functional groups of the as-prepared composites. The morphology and chemical composition of the composites were examined using field emission scanning microscopy (FESEM, JOEL JSM-T600F) and X-ray photoelectron spectroscopy (XPS, XSAMHS Kratos Analytical), respectively.

### Electrochemical analysis

The cyclic voltammetry (CV) and galvanostatic charge–discharge (GCD) analyses of individual electrodes were tested in a 1 M KCl solution via a three-electrode configuration. The performance of the assembled devices was further tested in a two-electrode configuration utilizing CV, GCD, electrochemical impedance spectroscopy (EIS) as well as a cycling stability test. The CV analyses were performed at different scan rates (5–100 mV/s) whereas GCD analyses were recorded at different current densities (1.0–2.0 A/g). EIS was conducted at a frequency range from 0.1 Hz to 10 kHz and a perturbation amplitude of 5 mV at an open circuit potential (OCP). The cycling stability test was measured over 4000 cycles.
